# Bridging the Gap Between Tolerogenic Dendritic Cells In Vitro and In Vivo: Analysis of Siglec Genes and Pathways Associated with Immune Modulation and Evasion

**DOI:** 10.3390/genes15111427

**Published:** 2024-10-31

**Authors:** Diahann T. S. L. Jansen, Tatjana Nikolic, Nicoline H. M. den Hollander, Jaap Jan Zwaginga, Bart O. Roep

**Affiliations:** Department of Internal Medicine, Leiden University Medical Center, 2333 ZA Leiden, The Netherlands; d.t.s.l.jansen@lumc.nl (D.T.S.L.J.); t.nikolic@lumc.nl (T.N.); j.j.zwaginga@lumc.nl (J.J.Z.)

**Keywords:** tolerogenic dendritic cells, Siglec, immune modulation, immune evasion

## Abstract

Background/Objectives: Dendritic cells (DCs) are master regulators of the adaptive immune response. Inflammatory DCs (inflamDCs) can prime inflammatory T cells in, for instance, cancer and infection. In contrast, tolerogenic DCs (tolDCs) can suppress the immune system through a plethora of regulatory mechanisms in the context of autoimmunity. We successfully generated tolDCs in vitro to durably restore immune tolerance to an islet autoantigen in type 1 diabetes patients in a clinical trial. However, cancers can induce inhibitory DCs in vivo that impair anti-tumor immunity through Siglec signaling. Methods: To connect in vivo and in vitro tolDC properties, we tested whether tolDCs generated in vitro may also employ the Siglec pathway to regulate autoimmunity by comparing the transcriptomes and protein expression of immature and mature inflamDCs and tolDCs, generated from monocytes. Results: Both immature DC types expressed most Siglec genes. The expression of these genes declined significantly in mature inflamDCs compared to mature tolDCs. Surface expression of Siglec proteins by DCs followed the same pattern. The majority of genes involved in the different Siglec pathways were differentially expressed by mature tolDCs, as opposed to inflamDCs, and in inhibitory pathways in particular. Conclusions: Our results show that tolDCs generated in vitro mimic tumor-resident inhibitory DCs in vivo regarding Siglec expression.

## 1. Introduction

Dendritic cells (DCs) are the instructors of adaptive T cell responses, capable of inducing both immunity and tolerance [1,2,3]. Inflammatory DCs (inflamDCs) can induce effector T cells in infection or cancer to clear pathogens or tumor cells, respectively. Conversely, tolerogenic DCs (tolDCs) can suppress the immune response and induce tolerance through, for instance, induction of antigen-specific regulatory T cells. This antigen-specific tolerance-inducing capacity is attractive as a therapeutic strategy for autoimmune diseases, where tolerance specifically towards disease-causing autoantigens is the goal [4,5,6].

In type 1 diabetes (T1D), tolerance to islet autoantigens is broken, causing autoimmune destruction of insulin-producing β-cells in the pancreatic islets of Langerhans by autoreactive T cells, resulting in insulin shortage and dysregulated blood glucose levels [7,8,9]. To restore immune tolerance to islet autoantigens, we generated tolDCs from monocytes using vitamin D3 and dexamethasone [10,11]. These tolDCs exhibited a plethora of regulatory mechanisms including inactivation of effector T cells, induction of antigen-specific regulatory T cells and transfer of tolerogenic properties to other DCs (infectious tolerance) [12,13,14,15,16,17,18,19]. In a successful phase 1 clinical trial, we showed that autologous tolDCs generated in vitro durably restored immune tolerance to islet autoantigens in T1D patients [10,11].

Strikingly, cancers are capable of inducing tolDCs in vivo to deter anti-tumor immunity. While the immune system is tasked with the detection and destruction of tumor cells, cancers employ elaborate mechanisms to modulate anti-tumor immunity and evade immune recognition [20,21]. Tumor cells are highly glycosylated with complex glycans. Sialic acids positioned at the end of these complex glycans are recognized by sialic acid-binding immunoglobulin-type lectins (Siglecs), which are found on the surface of most immune cells [22,23]. The Siglec family can be divided into two groups based on genetic homology among mammalian species or based on their function and intracellular signaling pathways. The majority of Siglecs (Siglec-2 to 12) bear an immunoreceptor tyrosine-based inhibitory motif (ITIM) or ITIM-like sequences in their cytoplasmic tail that recruit SHP-family phosphatases, resulting in inhibition of immune cell activation, comparable to the action of the immune checkpoint receptor PD-1. In contrast, Siglec-14, 15 and 16 are associated with an immunoreceptor tyrosine-based activation motif (ITAM) containing DAP12 through a positively charged lysine residue in the transmembrane domain, which can recruit PI3K and promote an inflammatory response through the MAPK and Akt pathways. Siglec-1 contains neither an ITIM nor an ITAM but is internalized upon ligand binding [22,23]. In a mouse model of melanoma, hypersialylation of tumor cells resulted in tumor growth associated with an increased regulatory/effector T cell ratio [22,24]. DCs that sampled sialylated antigens through Siglec-E (the murine homolog of Siglec-7 and Siglec-9) inhibited effector T cell function and induced regulatory T cells [25]. In mice, selective Siglec-E expression was observed in tumor-infiltrating DCs, leading to inhibition of their maturation, alteration of antigen processing and impairment of T cell activation. In human tumors, expression of inhibitory Siglec-7 and Siglec-9 was described on DCs in several types of cancer [26]. These results indicate that sialylation of tumor cells obstructs anti-tumor immunity through Siglec-induced tolDCs.

Previously, we reported on T1D risk genes in our in vitro-generated tolDCs and the reproducibility of their generation [27,28], but with insights from the tumor immunology field, we now focus on the Siglec family and wish to connect in vivo tolDCs’ properties with those of in vitro tolDCs by investigating whether in vitro-generated tolDCs can also employ the Siglec genes and pathways to regulate autoimmunity. Based on the diverse expression of inhibitory Siglecs by tolDCs generated in vitro, mimicking tumor-resident inhibitory DCs in vivo, Siglec signaling may be added to the inhibitory toolbox that tolDCs possess to induce tolerance.

## 2. Materials and Methods

### 2.1. Dendritic Cell Culture for Database Generation

TolDCs and inflamDCs were cultured as described previously [27,28]. In short, peripheral blood mononuclear lymphocytes (PBMCs) were isolated either from whole blood samples from T1D patients who consented to the study or from purchased buffy coats of anonymous healthy blood donors, followed by selection for CD14 positivity with CD14 microbeads (Miltenyi Biotec, Bergisch Gladbach, Germany). Monocytes were cultured in RPMI-1640 medium (Gibco, Waltham, MA, USA) supplemented with 8% FCS (Greiner Bio-One, Kremsmunster, Austria), glutamine and penicillin/streptomycin (Life technologies, Carlsbad, CA, USA), recombinant human IL-4 (500 U/mL, Invitrogen, Carlsbad, CA, USA) and recombinant human GM-CSF (800 U/mL, Invitrogen) for 6 days. To induce tolDCs, vitamin D3 (10^−8^ M, Dishman, Veenendaal, The Netherlands) and dexamethasone (10^−6^ M, Sigma-Aldrich, St. Louis, MO, USA) were added to the culture. On day 6, immature dendritic cells were harvested and used for RNA-seq analysis or matured using a mix of recombinant human cytokines: IL-1β (1600 U/mL), IL-6 (500 U/mL), TNF-α (335 U/mL) (all from Miltenyi Biotec, Bergisch Gladbach, Germany) and synthetic prostaglandin E2 (2 μg/mL; Pfizer, New York, NY, USA). Two days later, they were collected and used for RNA-seq.

**Figure 1:**
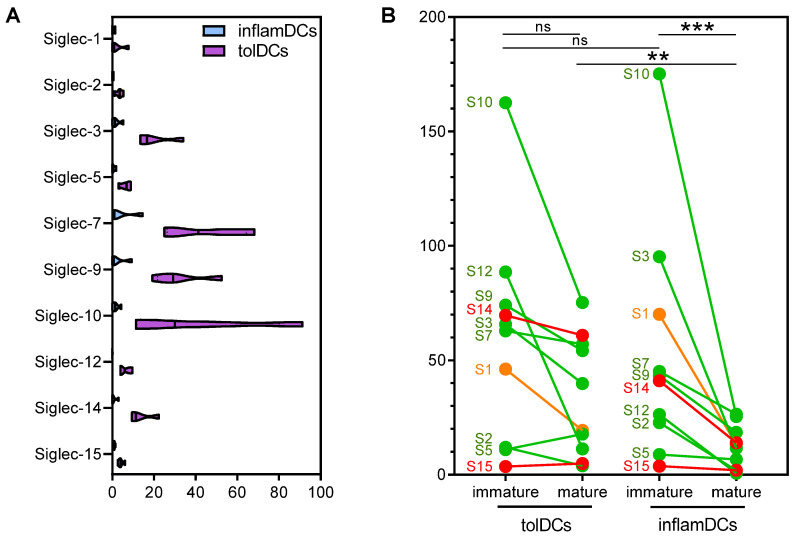
Siglec gene expression by immature and mature proinflammatory and tolerogenic dendritic cells. (**A**) Gene expression analysis of Siglecs with detectable RPKM in mature monocyte-derived dendritic cells (DCs). Monocytes were differentiated into either proinflammatory DCs (inflamDCs; blue) or tolerogenic dendritic cells using vitamin D3 and dexamethasone (tolDCs; purple) and matured with a mix of cytokines. n = 4. (**B**) To validate the results depicted in A, Siglec expression was determined in another set of cytokine-matured inflamDCs and tolDCs, including cells in the immature stage. Siglecs are divided based on their function: inhibitory (green), scavenging (orange) or activating (red). n = 11. Statistical analysis was performed using the Friedman test followed by Dunn’s test (**B**). ** *p* = 0.01; *** *p* = 0.002; ns non-significant.

Two separate datasets were used in this study; the first dataset consisted of mature inflamDCs and tolDCs from four healthy donors (Figure 1A), and the second (validation) dataset included eleven donors including six healthy controls and five T1D patients. The second dataset contained both immature and mature inflamDCs and tolDCs [27,28].

### 2.2. RNA Sequencing and Data Analysis

RNA was extracted from immature and mature DCs using a Quick-DNA/RNA Miniprep Kit (Zymo Research, Uden, Netherlands) following the manufacturer’s protocol. Details of the library preparation and RNA-seq data analysis have been described previously [27,28]. Genes with a false discovery rate (FDR) of less than 0.05 were considered significant.

### 2.3. Flow Cytometric Analysis

Siglec proteins’ surface expression was analyzed using the following antibodies: anti-Siglec-7 PerCP-Vio700 (clone REA214, Miltenyi Biotec, Leiden, The Netherlands), anti-Siglec-9 Alexa Fluor 594 (clone 191240, R&D Systems, Minneapolis, MI, USA) and anti-Siglec-10 Brilliant Violet 786 (clone 5G6, BD Biosciences, Franklin Lakes, NJ, USA). Cells were acquired on a BD LSRFortessa, and data were analyzed using FlowJo version 10.9.

### 2.4. Statistical Analysis

Data were analyzed and visualized with GraphPad Prism 8, the STRING database [29] and Cytoscape software (version 3.10.2). Either an unpaired Student’s *t*-test or ANOVA (Friedman test) was used to test statistical significance. A *p*-value of <0.05 was considered significant.

## 3. Results

### 3.1. High and Diverse Siglec Gene Expression in Immature Dendritic Cells Is Partially Retained by Mature Tolerogenic Dendritic Cells

To investigate whether in vitro-generated tolDCs can employ the Siglec pathway to regulate immune responses, we performed transcriptome analysis of monocyte-derived inflamDCs and tolDCs matured with a mix of cytokines from four healthy donors. Of the 15 Siglecs known to date, detectable reads per kilobase per million mapped reads (RPKM) were observed for 10 Siglecs in both inflamDCs and tolDCs (Figure 1A). Interestingly, Siglec expression was consistently higher in tolDCs. Specifically Siglec-3, Siglec-7, Siglec-9 and Siglec-10 were highly expressed by tolDCs, which are the Siglecs described to be expressed by monocyte-derived DCs [22].

To validate these results, RNA sequencing was performed in another set of monocyte-derived inflamDCs and tolDCs from 11 donors. To investigate the kinetics of Siglecs and answer the question of whether mature tolDCs increase their Siglec expression or retain high Siglec levels from the immature state, we also included immature inflamDCs and tolDCs. Immature inflamDCs and tolDCs expressed high and comparable levels of Siglecs (Figure 1B). This expression strongly declined in mature inflamDCs, but in mature tolDCs, the decline was less pronounced, resulting in significantly higher Siglec expression compared to mature inflamDCs (Figure 1B). We divided Siglecs based on their function, and again, the inhibitory Siglec-3, Siglec-7, Siglec-9 and Siglec-10 were highly expressed by mature tolDCs (Figure 1B, green). However, expression of the activating Siglec-14 was also retained by mature tolDCs (Figure 1B, red). Expression of the scavenging Siglec-1 declined in mature tolDCs, similar to mature inflamDCs (Figure 1B, orange).

Taken together, the results showed that mature tolDCs showed high gene expression of Siglec-3, Siglec-7, Siglec-9, Siglec-10 and Siglec-14, which they retained from the immature state, albeit in lower levels compared to immature tolDCs.

### 3.2. Siglec Protein Expression Largely Follows Siglec Gene Expression

**Figure 2:**
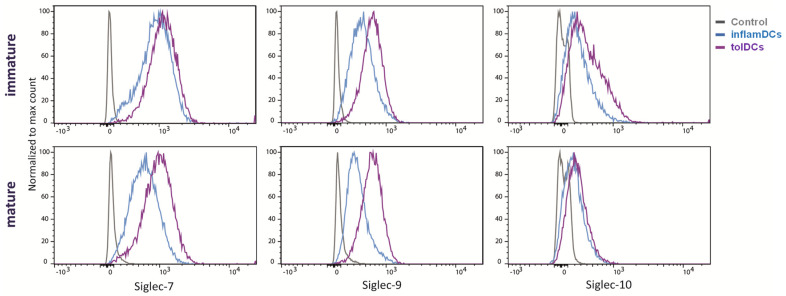
Siglec surface expression by immature and mature proinflammatory and tolerogenic dendritic cells. Siglec-7, Siglec-9 and Siglec-10 protein expression on the surface of both immature and mature proinflammatory and tolerogenic dendritic cells was analyzed using flow cytometry. The depicted histograms are overlays per Siglec of unstained control DCs (gray), proinflammatory DCs (inflamDCs; blue) and tolerogenic DCs (tolDCs; purple) of a representative donor.

Since RNA expression may not necessarily equal protein expression, we analyzed the surface protein expression of Siglec-7, Siglec-9 and Siglec-10, which showed the highest RNA expression in mature tolDCs (Figure 1A), on both immature and mature inflamDCs and tolDCs. Immature DC subsets showed comparable surface expression of all three Siglecs, which is in line with the RNA data (Figure 2). DC maturation using the cytokine mix resulted in decreased Siglec-7 and Siglec-9 surface expression by inflamDCs, while their expression persisted in tolDCs (Figure 2). Surface expression of Siglec-10 decreased upon maturation in both inflamDCs and tolDCs (Figure 2). Siglec-10 protein expression discorded with RNA quantity in immature DCs, which showed high RNA expression but low surface protein expression (Figure 1B and Figure 2). Thus, overall, immature DCs showed surface expression of Siglec-7 and Siglec-9, which was retained by mature tolDCs but decreased in mature inflamDCs, comparable to the RNA data.

### 3.3. Differentially Expressed Genes from the Siglec Interaction Networks Are Predominantly Associated with Inhibitory Siglecs and Enriched in the Immunoregulatory Interaction Network

**Figure 3:**
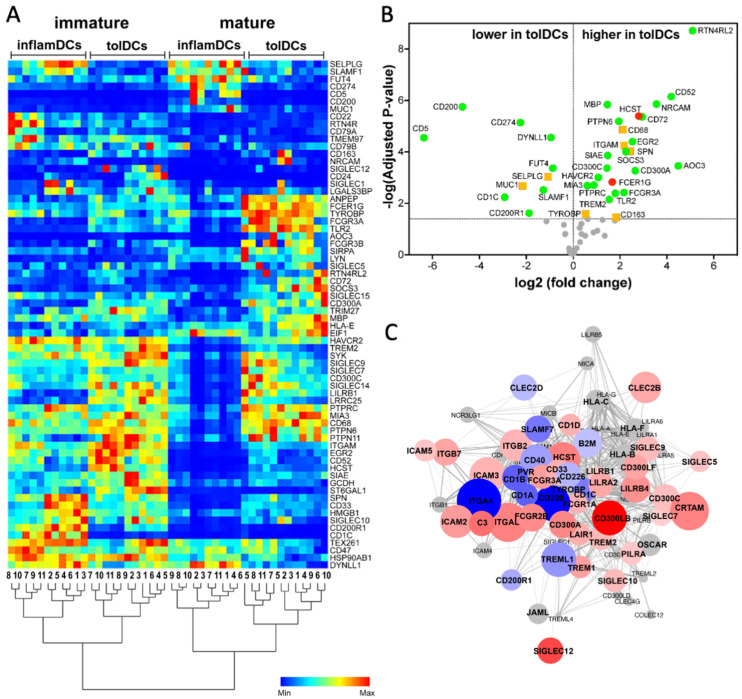
Differential gene expression of Siglec-related genes and their relationship in the immunoregulatory interaction pathway. (**A**) The heatmap shows unsupervised clustering of immature and mature tolDCs and inflamDCs generated from 11 independent donors using Siglecs and Siglec-related genes. (**B**) The volcano plot depicts log-transformed differential expression and adjusted significance values for 58 genes from the Siglec-related networks that are expressed by tolDCs and inflamDCs. The symbols depict genes associated with activating Siglecs only (red circles), inhibiting Siglecs only (green circles) and both activating and inhibiting Siglecs or Siglec1 (orange squares). (**C**) STRING interaction network of 78 genes expressed by tolDCs and inflamDCs within the Reactome pathway entitled ‘Immunoregulatory interactions between a Lymphoid and a non-Lymphoid cell’. The colors of the circles (nodes) represent the gene expression ratio between tolDCs and inflamDCs (LogFC): genes with higher expression in tolDCs are marked in shades of red, those with lower expression in tolDCs are marked in shades of blue and those with no difference in expression between tolDCs and inflamDCs are marked in gray. The size of each node is determined by the -Log (Adjusted *p*-value). For (**B**,**C**), the cut-off significance for differential expression was set at *p* < 0.05.

The effect of tolerogenic modulation on Siglec-associated proteins was explored by analyzing the expression of 95 genes from the Siglec-related STRING protein–protein interaction (PPI) networks in the transcriptomes of immature and mature tolDCs or inflamDCs. Of these 95 genes, 58 genes (61%) were expressed by both DC types. The gene expression heatmap and unsupervised clustering using Siglecs and Siglec-related genes clearly segregated mature from immature DCs, as well as tolDCs from inflamDCs (Figure 3A). As with Siglecs, differential expression of their related genes was more apparent between mature tolDCs and inflamDCs than between their immature progenitors. Among mature DCs, 38 out of 58 analyzed Siglec-related genes were significantly differentially expressed in tolDCs compared to inflamDCs (Figure 3B). Of these 38 genes, ten genes were expressed less by mature tolDCs, and the remaining 28 were expressed more by mature tolDCs compared to inflamDCs.

Regarding the relation of the differentially expressed genes with activating versus inhibiting Siglecs (Figure 3B), two genes (HCST and FCER1G) only interact with the activating Siglec-14 and Siglec-15, the TYROBP gene interacts with both activating and inhibiting Siglecs and 30 genes are associated with inhibiting Siglecs only. The remaining five genes (SPN, MUC1, ITGAM, CD68 and CD163) interact with Siglec-1. Notably, genes associating with inhibitory Siglecs predominantly had higher expression in tolDCs (21 genes), while nine genes had lower expression in tolDCs compared to inflamDCs.

In view of the multiple regulatory mechanisms that tolDCs can employ, we analyzed the enrichment of 46 differentially expressed Siglecs and Siglec-related genes in existing Reactome pathways (Table 1). This yielded significant results for eight pathways, of which HSA-198933 (‘Immunoregulatory interactions between a Lymphoid and a non-Lymphoid cell’) was most significantly enriched (strength 1.7, FDR = 4.5 × 10^−18^) and included 15 genes from our set. As the Siglecs and Siglec-interacting proteins are not the only immunoregulatory proteins that may be differentially expressed between tolDCs and inflamDCs, we extracted and analyzed the expression of all 130 genes from the HSA-198933 pathway (Figure 3C). When aligned with our gene expression data set, 78 immunoregulatory genes (of 130) from this pathway were expressed by dendritic cells, of which 51 (65%) were different between tolDCs and inflamDCs (16 with lower and 35 with higher expression in tolDCs than in inflamDCs).

In summary, the majority of Siglec-related genes are differentially expressed between tolDCs and inflamDCs and significantly represented in the immunoregulatory interaction pathway.

## 4. Discussion

Our study identifies Siglec signaling as candidate mechanism for tolerogenic DCs and immature DCs that are believed to have anti-inflammatory features, as compared to mature proinflammatory DCs. Thus, tolDCs generated in vitro share this immunomodulatory asset with tumor-resident tolerogenic DCs in cancer patients, and Siglec signaling can be added to the wide arsenal of immune inhibitory tools that in vitro-generated tolDCs possess to suppress adaptive immunity.

Data supporting Siglec signaling in tolDCs were derived from expression of Siglec receptors as well as increased expression of genes associated with or involved in Siglec signaling pathways. Siglec signaling may act in concert with other immunomodulatory features of tolDCs, including expression of MHC and other risk genes predisposing to T1D, production of suppressive cytokines and expression of immune-evasive defensins (e.g., cathelicidin) [28,30,31,32,33,34,35]. TolDCs can suppress the immune system by inactivating and deterring effector T cells, inducing regulatory T cells and conferring tolerogenic properties on other DCs (infectious tolerance) through a plethora of regulatory mechanisms [4,36], and it is conceivable that Siglec signaling contributes to some or all of these mechanisms.

TolDCs have been at the focus of attention owing to their potential to serve as advanced medicinal cell therapy to intervene in autoimmune diseases. We have recently assessed their candidacy as immune intervention therapy in T1D [5,10,11]. Our preclinical studies demonstrated mechanisms of action supporting these hypotheses. In humanized HLA-DR4-transgenic mice, proinsulin peptide–pulsed tolDCs prevented and reversed induced autoimmunity to proinsulin, and the effect lasted upon subsequent challenges with the islet autoantigen [37]. Human tolDCs regulate adaptive immunity by antigen-specifically eliminating CD4^+^ and CD8^+^ T cells and inducing antigen-specific Tregs [12,13,14,37,38]. Tregs, in turn, change mature DCs to become anti-inflammatory (‘infectious tolerance’) and suppress immune responses to other islet autoantigens present on the same DC (‘linked suppression’) [13,16]. These processes proved to be critically dependent on expression of PD-L1, membrane-bound TNF, ICOS-L, B7-H3 and the appropriate HLA on tolDCs to allow antigen specificity. TolDC-induced Tregs resemble induced antigen-specific Tr-1, in vivo circulating islet-specific Tregs and (activated) thymus-derived Tregs (tTregs) [19,39,40]. In a placebo-controlled, dose-escalation phase 1 clinical trial in adult patients with long-standing T1D, we demonstrated the safety and feasibility of two vaccinations (prime and boost) with tolDCs pulsed with a proinsulin peptide and identified immune correlates of the mechanistic immunological efficacy of intradermally injected tolDCs [10,11]. The tolDC vaccine induced a profound and durable decline in pre-existing autoimmune responses to the vaccine peptide up to three years after therapy and a temporary decline in CD4^+^ and CD8^+^ T cell responses to other islet autoantigens. While major leukocyte subsets remained stable, ICOS^+^CCR4^+^TIGIT^+^ Tregs and CD103^+^ tissue-resident and CCR6^+^ CD4^+^ effector/memory T cells increased in response to the first tolDC injection. Notwithstanding the encouraging clinical results of inverse vaccination with autoantigen-loaded tolDCs, clinical cell therapy is challenging, labor-intensive and expensive, and it requires the generation of a personalized cell product. While the manufacturing process was proven to be reproducible and transferable between international institutes [27] and adequate release criteria have been defined and validated [11,19], it would seem preferable to develop an off-the-shelf vaccine that can selectively target tolDCs and their progenitors in vivo. In light of the present study, we believe that inhibitory Siglecs offer the potential to selectively target autoantigens that have been engineered to serve as ligands of Siglecs expressed on tolDCs in vivo to ensure induction of autoantigen-specific immune suppression and avoid exacerbation of proinflammatory autoimmunity. Indeed, we previously demonstrated that ex vivo and in vivo DC targeting of the α2-3 sialic acid–modified antigen to the mouse homolog of human Siglec-9 (i.e., Siglec-E) drove naive CD4^+^ T-cells to differentiate into antigen-specific Treg cells, while DCs treated with the sialic acid–modified antigen dampened T cell differentiation to effector T cells even in the presence of native antigen-loaded DCs [25]. We also showed that a sialylated mite allergen bound to Siglec-9 present on monocytes and DCs and led to suppressed CD4^+^ T cell activation and diminished production of T_H_2 cytokines, whereas the native mite allergen aggravated allergic immunity [41]. Our results warrant exploration of this ‘inverse’ vaccination strategy in the context of autoimmune diseases.

In summary, we showed that tolDCs generated in vitro express high RNA and protein levels of Siglecs with inhibitory capacities mimicking those of in vivo inhibitory DCs found in the tumor environment.

## Figures and Tables

**Table 1:** Significantly enriched Reactome pathways containing the 46 differentially expressed Siglec and Siglec-related genes.

Pathway	Gene Count		Enrichment Strength	FDR
	Observed	Total		
Immunoregulatory interactions between Lymphoid and non-Lymphoid cells	15	130	1.7	4.5 × 10^−18^
Immune System	30	1979	0.8	3.1 × 10^−16^
Adaptive Immune System	21	758	1.1	5.3 × 10^−15^
Neutrophil degranulation	14	476	1.1	1.9 × 10^−9^
Innate Immune System	18	1041	0.9	3.6 × 10^−9^
Other semaphorin interactions	4	19	2.0	8.4 × 10^−5^
DAP12 interactions	4	39	1.6	9.7 × 10^−4^
Cell surface interactions at the vascular wall	5	139	1.2	5.4 × 10^−3^

## Data Availability

The databases presented in this study have been published previously and are available on request from the corresponding author of the two studies.
